# Supplementary material for: Biochemical principles of miRNA targeting in flies
Source: bioRxiv. 2024 Nov 16:2024.11.16.623948. Preprint. [Version 1] doi: 10.1101/2024.11.16.623948 (PMC11601291; doi:10.1101/2024.11.16.623948)

bioRxiv preprint doi: https://doi.org/10.1101/2024.11.16.623948; this version posted November 16, 2024. The copyright holder for this preprint (which was not certified by peer review) is the author/funder, who has granted bioRxiv a license to display the preprint in perpetuity. It is made available under aCC-BY 4.0 International license.

Supplementary Figure Titles and Legends

Fig. S1 | The effect of G:U pairs within the seed on miRNA binding affinity. **(a)** Equilibrium binding assays of Ago1•*let-7* for seed-matching and fully complementary target RNA using double-filter binding assay. **(b)** K_D values for canonical seed-matched sites (left: t2–t8 targets with t1A; right: t2–t8 targets) and one, two and three G:U pairs within *bantam*, miR-11, and miR-184 seed at indicated positions. Shown for comparison: 8mer (cyan), 7mer-m8 (orange), 7mer-A1 (blue), 6mer-A1 (purple) and 6mer-m8 (gray). Error bars indicate 95% CI on the median.

Fig. S2 | Structures of Ago1•miR-184 bound to t3-, t4-, and t5-mismached 8mer sites predicted by AlphaFold 3.

Fig. S3 | Structures of Ago1•*let-7* bound to t3-, t4-, and t5-mismached 8mer sites predicted by AlphaFold 3.

Fig. S4 | Ago1 cleaves centered sites in vitro. **(a)** Uncropped image of polyacrylamide gel electrophoresis shown in **Fig. 5b**. Sequence-specific cleavage was blocked when the target RNA contained a phosphorothioate linkage (orange) flanked by 2′-*O*-methyl ribose at positions t10 and t11 (red). **(b)** Ago1•*let-7* cleaved both centered sites at levels comparable to those observed with a fully complementary RNA. Cleavage reactions were performed with 3.5 nM Mg^{2+} and contained 0.32 nM Ago1•*let-7* and 5 nM **(a)** or 100 nM **(b)** RNA target.

bioRxiv preprint doi: https://doi.org/10.1101/2024.11.16.623948; this version posted November 16, 2024. The copyright holder for this preprint (which was not certified by peer review) is the author/funder, who has granted bioRxiv a license to display the preprint in perpetuity. It is made available under aCC-BY 4.0 International license.

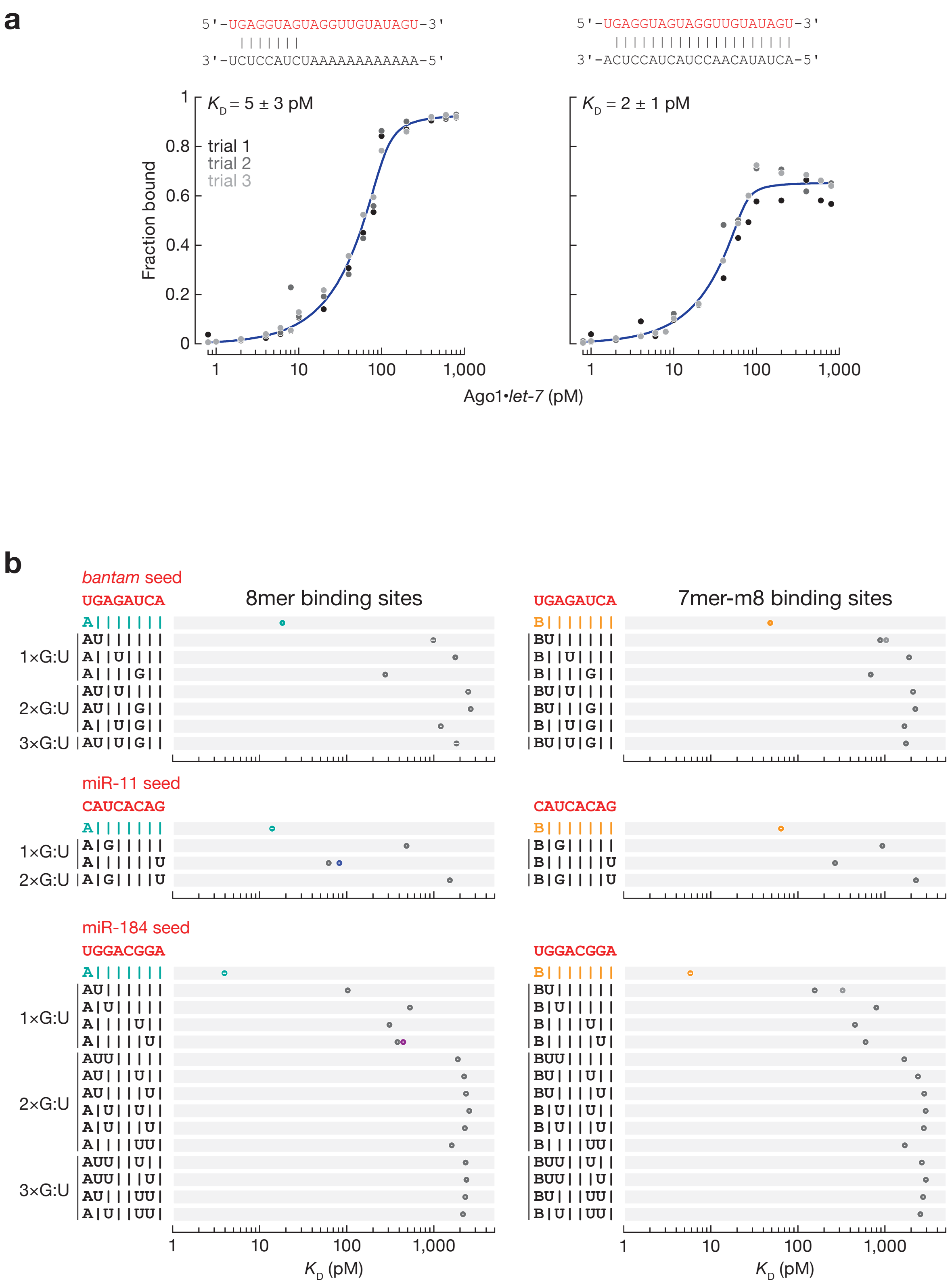

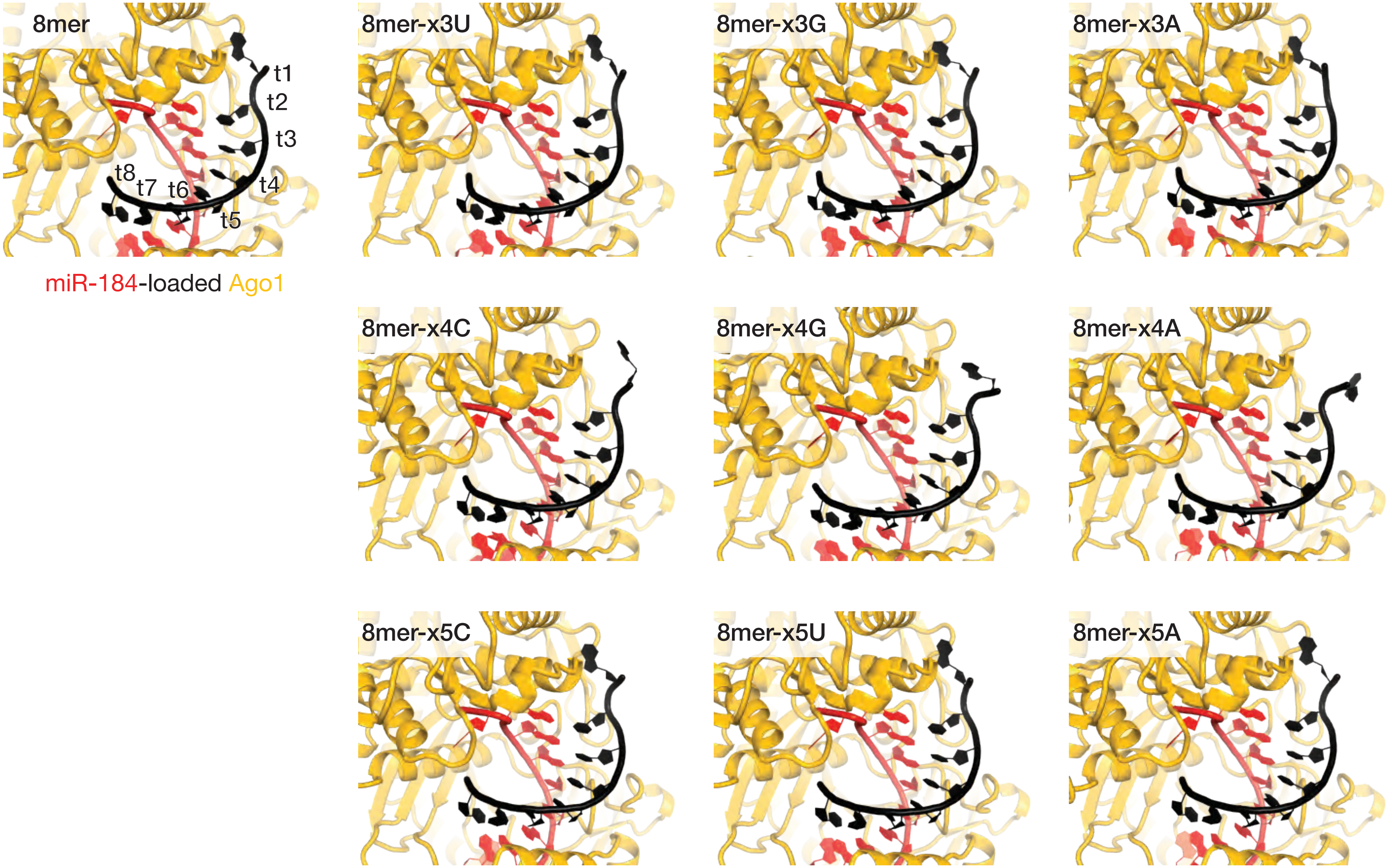
8mer
t1
t2
t3
t4
t5
t6
t7
t8
miR-184-loaded Ago1
8mer-x3U
8mer-x3G
8mer-x3A
8mer-x4C
8mer-x4G
8mer-x4A
8mer-x5C
8mer-x5U
8mer-x5A

Vega-Badillo et al., Fig. S3

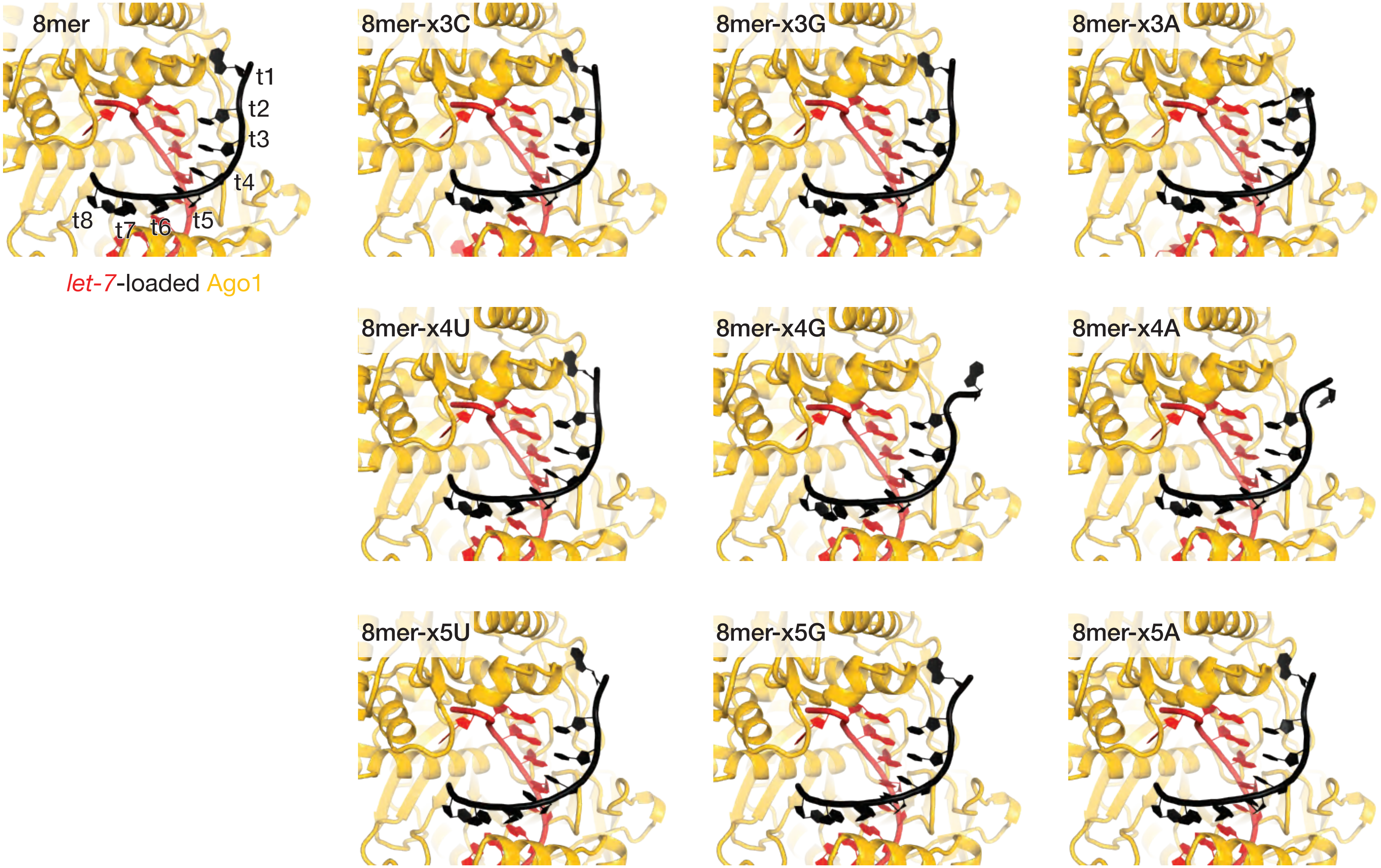

bioRxiv preprint doi: https://doi.org/10.1101/2024.11.16.623948; this version posted November 16, 2024. The copyright holder for this preprint (which was not certified by peer review) is the author/funder, who has granted bioRxiv a license to display the preprint in perpetuity. It is made available under aCC-BY 4.0 International license.

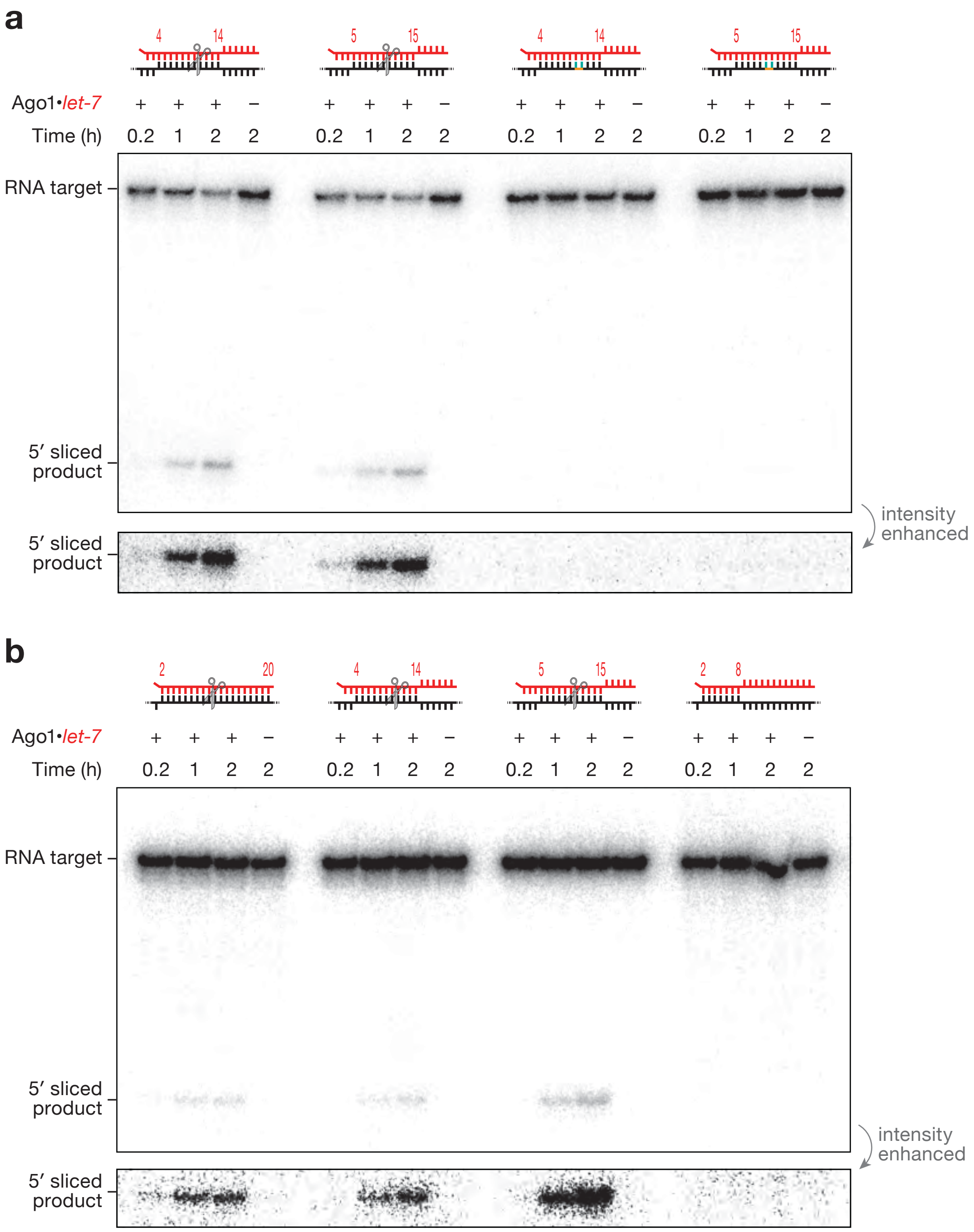

Supplement: 2 [file NIHPP2024.11.16.623948v1-supplement-2.pdf]
